# Chronically stressed or stress-preconditioned neurons fail to maintain stress granule assembly

**DOI:** 10.1038/cddis.2017.199

**Published:** 2017-05-11

**Authors:** Tatyana A Shelkovnikova, Pasquale Dimasi, Michail S Kukharsky, Haiyan An, Annamaria Quintiero, Claire Schirmer, Luc Buée, Marie-Christine Galas, Vladimir L Buchman

**Affiliations:** 1 School of Biosciences, Cardiff University, Cardiff, UK; 2 Institute of Physiologically Active Compounds Russian Academy of Sciences, Chernogolovka, Moscow Region, Russian Federation; 3 University Lille, Inserm, CHU-Lille, UMRS1172, Alzheimer & Tauopathies, Lille, France

## Abstract

Dysregulation of stress granules (SGs) and their resident proteins contributes to pathogenesis of a number of (neuro)degenerative diseases. Phosphorylation of eIF2*α* is an event integrating different types of cellular stress and it is required for SG assembly. Phosphorylated eIF2*α* (p-eIF2*α*) is upregulated in the nervous system in some neurodegenerative conditions. We found that increasing p-eIF2*α* level by proteasomal inhibition in cultured cells, including mouse and human neurons, before a SG-inducing stress (‘stress preconditioning’), limits their ability to maintain SG assembly. This is due to upregulation of PP1 phosphatase regulatory subunits GADD34 and/or CReP in preconditioned cells and early decline of p-eIF2*α* levels during subsequent acute stress. In two model systems with constitutively upregulated p-eIF2*α*, mouse embryonic fibroblasts lacking CReP and brain neurons of tau transgenic mice, SG formation was also impaired. Thus, neurons enduring chronic stress or primed by a transient mild stress fail to maintain p-eIF2*α* levels following subsequent acute stress, which would compromise protective function of SGs. Our findings provide experimental evidence on possible loss of function for SGs in certain neurodegenerative diseases.

Stress granules (SGs) are cytoplasmic RNA–protein macrocomplexes that form as a normal cellular response to a moderate-to-severe stress and serve to protect cellular RNAs from degradation until translation can be safely restored.^1^ SGs are believed to be protective under stress not only because they guard sensitive transcripts, but also because they participate in signaling events including anti-apoptotic signals,^2, 3, 4^ favor translation of molecular chaperones/other cytoprotective proteins^1^ and help adjusting cellular translation rates to accumulation of misfolded proteins that accompany some types of stress.^5^

A growing list of SG proteins have been implicated in neurodegenerative conditions, primarily amyotrophic lateral sclerosis (ALS) and a related condition, frontotemporal lobar degeneration (FTLD); many of them are modified by mutations in the encoding genes in these diseases. Prominent examples are TDP-43 and FUS, and mutations affecting SG proteins TAF15, EWS, hnRNP A2/B1, hnRNP A1, TIA-1, profilin1, ataxin-2, angiogenin, Gle1 and CREST have also been reported in rare cases of familial and sporadic neurodegenerative proteinopathies.^6, 7, 8, 9, 10^ There is also evidence of secondary dysregulation of SG proteins in Alzheimer’s disease and other tauopathies.^11, 12^ However, it still remains to be established whether neurons suffer from toxicity of persisting SG, for example, because SGs become seeds for pathological aggregation of RNA-binding proteins, or rather from loss of SG function because of their impaired assembly.

SG formation is tightly linked to phosphorylation of the translation initiation factor eIF2*α*. The latter event depletes the eIF2/tRNAiMet/GTP ternary complex required for translation initiation causing translational arrest, polysome disassembly and eventually SG assembly.^13^ Increased levels of p-eIF2*α* have been detected in the nervous system of patients with some neurodegenerative conditions such as Alzheimer’s disease and prion disorders,^14, 15, 16^ as well as animal models of neurodegeneration.^17, 18, 19, 20^ Although SG formation in most cases requires elevated p-eIF2*α* levels, it is still not clear how SG formation would be modulated by the presence of increased ‘basal’ (pre-stress) p-eIF2*α* levels. The answer to this question is crucial for understanding how neurons under conditions of developing proteinopathy and hence chronically activated stress response would respond to an acute, SG-inducing stress.

Here we report that, counterintuitively, short-term or chronic elevation of p-eIF2*α* levels, when coupled with upregulation of its phosphatase, impairs the ability of cells, including neurons *in vitro* and *in vivo*, to maintain SG assembly following a SG-inducing stress.

## Results

### Stress preconditioning by proteasome inhibition impairs SG assembly following acute stress

**Figure 1:**
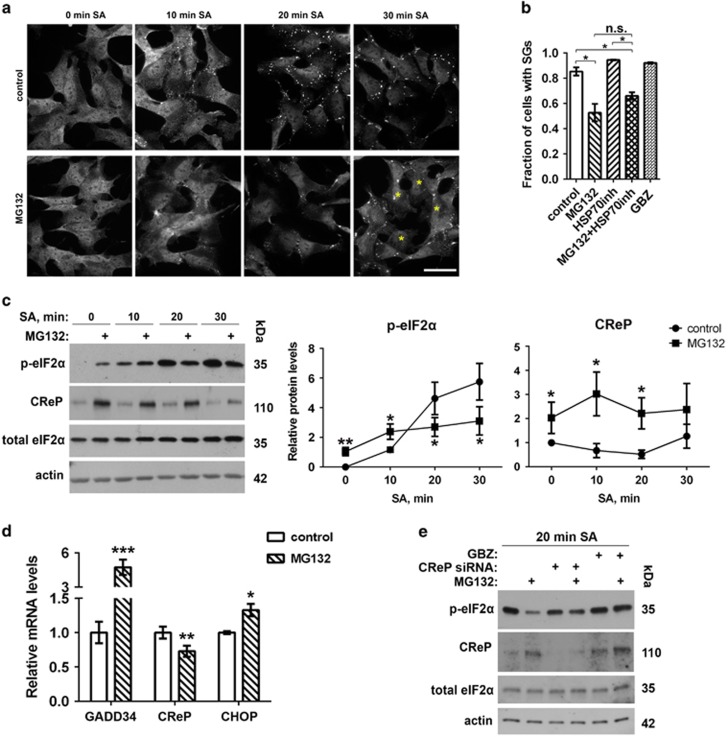
Stress preconditioning with a proteasome inhibitor MG132 upregulates eIF2*α* phosphatase regulatory subunits and limits the ability of SH-SY5Y cells to maintain p-eIF2*α* levels and SG assembly during stress. (**a**) SG assembly during oxidative stress is impaired after MG132 pretreatment. Cells were pretreated with MG132 (1 *μ*M for 4 h) or left untreated, and SA was added for 10, 20 or 30 min. SGs were visualized with anti-TIAR staining. Representative images of all conditions are shown, asterisks indicate cells without SGs. Scale bar, 10 *μ*m. (**b**) MG132 pretreatment leads to reduced number of cells with mature SGs after 30 min of SA exposure (*n*=3; *P<0.05). In contrast, pretreatment with a GADD34 phosphatase subunit inhibitor guanabenz does not affect SG assembly during stress. Cells were pretreated with 1 *μ*M MG132 for 4 h or 5 *μ*M guanabenz for 24 h. HSP70 inhibitor pifithrin (5 *μ*M) was added 30 min before SA addition. (**c**) MG132-pretreated and control cells display different kinetics of p-eIF2*α* build-up during stress, and CReP protein is upregulated by MG132. Cells were treated with MG132 and subsequently stressed with SA as described in (**a**). Representative blots and quantification of band intensities for p-eIF2*α* and CReP in MG132-pretreated as compared with control cells are shown (*n*=5 or 6, *P<0.05, **P<0.01). For CReP and p-eIF2*α* band analysis, 0 and 10-min band intensity in control cells were taken as 1, respectively. (**d**) MG132 upregulates GADD34 mRNA. Cells were treated with 1 *μ*M MG132 for 4 h before RNA extraction for qRT-PCR analysis. CHOP mRNA levels were measured in parallel to confirm activated stress response in MG132-treated cells (*n*=5, *P<0.05, **P<0.01, ***P<0.001). (**e**) Pharmacological inhibition of GADD34 and to a lesser extent CReP knockdown reverse the effect of MG132 pretreatment on p-eIF2*α* levels during stress. Cells were transfected with CReP siRNA and 72 h post-transfection treated with 1 *μ*M MG132 for 4 h followed by SA stress; or co-treated with MG132 and 50 *μ*M guanabenz for 4 h followed by SA stress. Cells were analyzed 20 min after SA addition. Representative western blots are shown

Dysfunction of the proteasome can induce stress response and has long been known to contribute to neurodegeneration.^21^ Treatment of SH-SY5Y neuroblastoma cells with a low concentration of a proteasomal inhibitor MG132 moderately increases p-eIF2*α* levels but does not induce SG assembly (Supplementary Figures S1a and b). We therefore used this treatment as stress ‘preconditioning’, to elevate p-eIF2*α* levels without concomitant SG assembly, and assessed the dynamics of SG formation and p-eIF2*α* levels after subsequent strong acute stress. SH-SY5Y cells were pretreated with 1 *μ*M MG132 for 4 h followed by exposure to 0.5 mM sodium arsenite (SA). Visible SGs appeared in both MG132-pretreated and control cells 10 min after SA addition, however, after 20 min, fewer MG132-pretreated cells displayed SGs, and after 30 min, the fraction of MG132-pretreated cells that developed mature SGs was significantly lower as compared with control cells (Figures 1a and b). This effect was not due to the ability of MG132 to stimulate HSP70 expression,^22^ as co-treatment with an HSP70 inhibitor prifithrin *μ*^23^ did not prevent the effect of MG132 on SG assembly (Figure 1b). The difference in SG assembly rate correlated with the difference in the dynamics of eIF2*α* phosphorylation during SA treatment: in MG132-pretreated cells, p-eIF2*α* levels, although higher than in control cells before and soon after SA addition (0- and 10-min SA, correspondingly), failed to increase further and remained lower than in control cells later on (Figure 1c). Thus, MG132-pretreated cells fail to uphold p-eIF2*α* levels during an acute stress at a level appropriate for maintaining SG assembly.

Dephosphorylation of p-eIF2*α* requires enzymatic activity of PP1 phosphatase catalytic subunit, which is modulated by one of the two regulatory subunits, CReP or GADD34. CReP is expressed constitutively and maintains low basal p-eIF2*α* levels, whereas GADD34 is the stress-inducible subunit. We hypothesized that MG132 pretreatment increases GADD34 and/or CReP levels leading to accelerated p-eIF2*α* dephosphorylation during stress. Indeed, GADD34 mRNA was markedly upregulated by MG132 (Figure 1d). We did not find a commercially available antibody, which would reliably recognize human GADD34 protein, but as GADD34 mRNA is known to be rapidly translated under stress,^24^ high GADD34 protein levels can be anticipated in these cells. Furthermore, although CReP mRNA level was slightly decreased (Figure 1d), we detected significant accumulation of CReP protein in the presence of MG132, which was maintained throughout SA treatment (Figure 1c). This result was likely due to impaired degradation of CReP, a protein with relatively short, ~2 h, half-life,^25^ when proteasome is inhibited. To directly address whether GADD34 and/or CReP activation is responsible for p-eIF2*α* downregulation during stress in MG132-pretreated cells, we used a selective small molecule GADD34 inhibitor, guanabenz,^26, 27^ and siRNA-mediated CReP knockdown. Cells were either transfected with CReP siRNA and treated with MG132 for 4 h or co-treated with MG132 and guanabenz for 4 h, this was followed by treatment with SA for 20 min. Guanabenz rescued both MG132-induced decrease of p-eIF2*α* levels and SG assembly, whereas CReP knockdown had a less pronounced effect (Figure 1e,Supplementary Figure 1c). We also noted that p-eIF2*α* levels were decreased in stressed CReP siRNA-transfected cells, which was likely due to compensatory induction of GADD34 (Supplementary Figure 1d). In contrast to MG132, 24-h pretreatment with 5 *μ*M guanabenz (an optimized concentration, which increases p-eIF2*α* levels but neither upregulates phosphatase regulatory subunits nor induces SGs in SH-SY5Y cells, data not shown) did not lead to impaired SG assembly during SA stress (Figure 1b).

These data demonstrate that stress preconditioning by proteasome inhibition leads to upregulation of PP1 phosphatase regulatory subunits thereby attenuating stress-induced p-eIF2*α* build-up during acute stress and negatively affecting SG assembly.

### SG assembly is impaired after stress preconditioning in cultured mouse and human neurons

To validate these findings in more relevant cells, we first examined the response of cultured mouse hippocampal neurons to stress preconditioning. As a result of the lower threshold for SG assembly in these cells in response to MG132 as compared with SH-SY5Y cells, an optimized concentration (200 nM MG132) was used. SA treatment for 70 min led to prominent upregulation of p-eIF2*α* and efficient SG formation in >80% of neurons, however, with MG132 pretreatment, the fraction of neurons that developed SGs was significantly decreased (Supplementary Figure 2).

**Figure 2:**
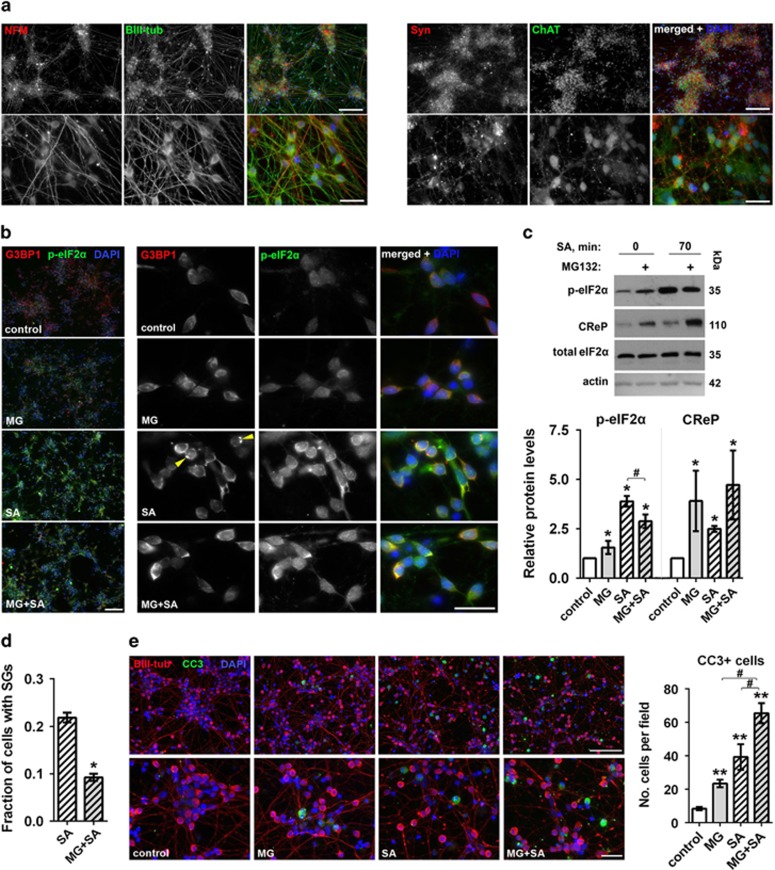
Stress preconditioning of human ES-derived neurons impairs their ability to maintain p-eIF2*α* levels and SG assembly. (**a**) Characterization of differentiated human neurons used in the study. Day 40 cultures of neurons differentiated from hES cells express typical neuronal markers *β*III-tubulin, synaptophysin and neurofilament M, as well as a motor neuron marker ChAT. (**b**) G3BP1-positive SGs and increased level of p-eIF2*α* in human neurons after 70 min of exposure to SA. Left column shows general plane images of treated and untreated cultures. Representative images are shown. Arrowheads indicate cells with SGs. (**c**) Pretreatment with MG132 upregulates CReP protein and results in attenuated build-up of p-eIF2*α* following SA stress as compared with control neurons. Representative western blots and quantification of band intensities are shown (*n*=4, * and ^#^*P*<0.05, asterisks indicate significant difference as compared with control cells). (**d**) Pretreatment with MG132 reduces the fraction of human neurons with SGs following SA stress. Fraction of neurons with G3BP1-positive SGs in was quantified (~400 neurons per condition, *P<0.05). (**e**) MG132 pretreatment decreases survival of human motor neurons during oxidative stress. MG132-pretreated and control cultures were treated with SA for 70 min and the number of cleaved caspase 3 (CC3)-positive cells was quantified from three independent experiments as described in Materials and methods section (^#^*P*<0.05, **P<0.01, asterisks indicate significant difference as compared with control cells). Representative images for all conditions are also shown. Motor neuron cultures were pretreated with 1 *μ* MG132 for 4 h in all panels. Scale bars, 200 *μ*m for top panels in (**a**), left column in (**b**) and top panel in (**e**); and 10 *μ*m for bottom panel in (**a**), right columns in (**b**) and bottom panel in (**e**)

To verify that human neurons respond to stress preconditioning in a similar way, we used human ES cells-derived motor neurons. The majority of neurons in day 40 neuronal cultures, which were used for experiments, displayed typical neuronal morphology; expressed established neuron markers including *β*III-tubulin, neurofilament M, synaptophysin and a motor neuron-specific marker ChAT (Figure 2a); and were functional as defined by electrophysiological recordings (Supplementary Figures 3a-c). As TIAR-positive dots were observed in the soma of the majority of naive human neurons masking SGs (Supplementary Figure 3d), for analysis of SGs in human neurons we used another established SG marker, G3BP1, which gave a diffuse staining pattern in untreated cells (Figure 2b). Similar to neuroblastoma cells, MG132 pretreatment increased p-eIF2*α* and CReP levels in human neurons (Figures 2b and c). Surprisingly, the concentration of SA that induced SGs in virtually all SH-SY5Y cells and the majority of hippocampal neurons, led to SG assembly in only ~20% of neurons even after 70 min of treatment (Figures 2b and d). Nevertheless, similar to mouse neurons, the fraction of cells with SGs among MG132-pretreated neurons was significantly smaller compared with control cultures (Figure 2d). In line with this, p-eIF2*α* levels were significantly lower in MG132-treated cells after 70 min of SA (Figure 2c). We did not observe GADD34 mRNA upregulation (data not shown) indicating that CReP may be mainly responsible for p-eIF2*α* dephosphorylation in this experimental system. Importantly, failure to maintain p-eIF2*α* levels/SG assembly was associated with decreased viability of human neurons – we detected significantly more cleaved caspase 3-positive neurons in MG132-pretreated cultures after 70 min of SA exposure (Figure 2e).

### Constitutive upregulation of p-eIF2*α* also results in reduced ability to maintain p-eIF2*α* levels and SG assembly during stress

Certain neurodegenerative conditions, such as Alzheimer’s disease, are characterized by increased levels of p-eIF2*α* in the brain;^16^ this can be a response to accumulation of misfolded proteins, proteasome inhibition and mild oxidative stress.^28^ It is feasible that such conditions of chronically increased eIF2*α* phosphorylation may affect SG formation. To test this, we first used embryonic fibroblasts from mice with germline inactivation of the gene encoding CReP (CReP KO MEFs).^29^ Their use allowed excluding possible side-effects of specific preconditioning agents and ensured constitutive p-eIF2*α* upregulation.

**Figure 3:**
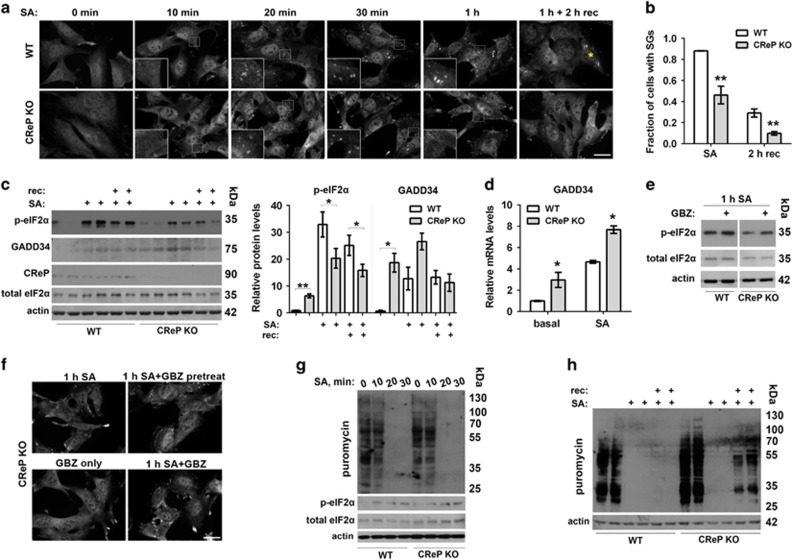
CReP KO MEFs characterized by chronically elevated basal p-eIF2*α* levels are deficient in SG assembly. (**a** and **b**) Initial stages of SG assembly are not affected but SG assembly cannot be maintained through the entire duration of stress in CReP KO cells. WT and CReP KO MEFs were treated with SA for the indicated times. Representative images of TIAR-positive SGs are shown. An asterisk indicates a cell with SGs after 2 h of recovery (**a**). The fraction of cells with mature SGs was quantified immediately after 1 h of SA and after 2 h of recovery (*n*=3, **P<0.01) (**b**). Scale bar, 10 *μ*m. (**c**) CReP KO MEFs display elevated basal p-eIF2*α* but fail to maintain high p-eIF2*α* levels during stress and recovery. Cells were treated with SA for 1 h or left to recover for 30 min after SA wash-off. Representative western blots and band intensity quantification are shown (*n*=6, *P<0.05, **P<0.01). (**d**) GADD34 mRNA level is increased in CReP KO MEFs at basal state and after 1 h of SA (*n*=4, *P<0.05). (**e**) Guanabenz prevents early decline in p-eIF2*α* levels post-stress in CReP KO MEFs. WT and CReP KO cells were stressed with SA for 1 h and guanabenz (GBZ, 50 *μ*M) was added together with SA. A representative western blot is shown. (**f**) Guanabenz rescues SG assembly in CReP KO cells. Cells were pretreated with guanabenz (50 *μ*M) for 30 min before SA addition (1 h SA + GBZ pretreat) or guanabenz was added to the cells together with SA (1 h SA+GBZ). Note that guanabenz alone does not induce SGs. Representative images of all conditions are shown. Scale bar, 10 *μ*m. (**g**) Shutdown of translation and the build-up of p-eIF2*α* during SA-induced stress occur normally in CReP KO MEFs. Cells were treated with SA for the indicated times, and newly synthesized proteins were labeled with puromycin and analyzed by western blot. (**h**) Translational recovery is accelerated in CReP KO fibroblasts. Cells were treated with SA and left to recover for 30 min. Note the appearance of puromycilated proteins in CReP KO cells but not WT cells after 30 min of recovery. In (**g** and **h**), representative blots of the experiments repeated three times are shown

SG assembly was not visibly affected in CReP KO cells as compared with isogenic WT MEFs during the first 30 min of SA treatment (Figure 3a). However, significantly fewer CReP KO cells contained SGs after 1 h of SA stress and after 2 h of recovery (Figures 3a and b). In line with this, despite elevated p-eIF2*α* levels in naive CReP KO cells, its levels in stressed CReP KO cells were lower than in WT cells 1 h after SA addition and during recovery (Figures 3c and d). Upregulation of the stress-inducible phosphatase subunit GADD34 was observed in naive and SA-stressed CReP KO MEFs as compared with WT MEFs (Figures 3c and d). This functional compensation for the absence of CReP could explain the ability of these cells to prematurely bring down p-eIF2*α* level and the observed failure to maintain SG assembly. To verify this experimentally, we inhibited the activity of GADD34 in CReP KO MEFs using guanabenz and assessed SG formation and p-eIF2*α* levels. Co-treatment with guanabenz indeed rescued impaired SG assembly and p-eIF2*α* levels in these cells after 1 h of SA (Figures 3e and f).

SG formation is coupled to suppression of protein translation. Using puromycin incorporation assay, we showed that, consistent with normal onset of SG assembly in CReP KO cells (Figure 3a, 20 min SA), translational shutdown occurred simultaneously in WT and KO cells, and the rate of p-eIF2*α* build-up was also similar in both cell lines up to 30 min of SA exposure (Figure 3g). However, CReP KO cells restored translation much earlier than WT cells – we were able to detect the appearance of puromycilated proteins already after 30 min of recovery (Figure 3h). These results indicate that similar to chemically preconditioned cells, in cells with chronic upregulation of p-eIF2*α*, intrinsically high activity of the phosphatase complex can also lead to early p-eIF2*α* decline, restoration of translation and inability to maintain SG assembly throughout stress.

### Neurons in a transgenic mouse model of tauopathy characterized by elevated levels of p-eIF2*α* are deficient in SG assembly

**Figure 4:**
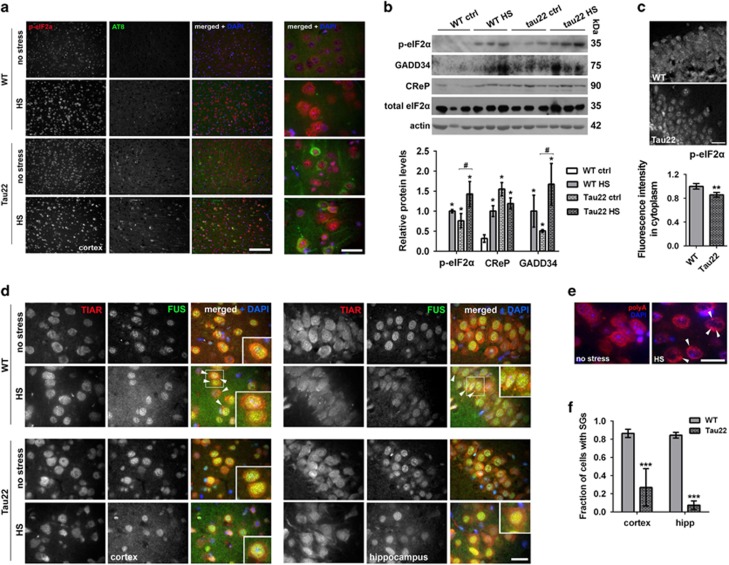
Transgenic mouse model of tauopathy is characterized by elevated basal p-eIF2*α* levels and impaired SG assembly in the brain. (**a**) p-eIF2*α* immunoreactivity is increased in the cortex of 8-month-old Tau22 transgenic animals compared with age-matched wild type (WT) controls under normal conditions and after HS. AT8 antibody was used to visualize phospho-tau on sagittal brain sections. (**b**) Levels of p-eIF2*α*, CReP and GADD34 proteins are higher in the hippocampus (CA1 subfield) of Tau22 animals compared with age-matched WT control animals (WT ctrl) and are upregulated by HS. Representative western blots and band intensity quantitation are shown (* and ^#^*P*≤0.05, asterisks indicate significant difference as compared with naive WT animals). For all proteins, band intensity for WT animals after HS was taken as equal 1. (**c**) Decreased p-eIF2*α* intensity in the cytoplasm of hippocampal neurons of Tau22 mice after HS. Representative images of p-eIF2*α* staining are shown for the same animals as in **a**. Details of fluorescence intensity analysis are given in Materials and methods section (**P<0.01). (**d**-**f**) Efficient HS-induced SG assembly is observed in cortical and hippocampal neurons of WT mice but not in Tau22 animals. SGs (arrowheads) were visualized using anti-TIAR antibody (**d**) or RNA-FISH with a fluorescently labeled poly(dT) probe (**e**); in (**d**), co-staining with an anti-FUS antibody in was used to highlight nuclei. For quantification of SG-containing cells, >100 neurons were assessed in each of the studied regions in three HS-treated mice per genotype (***P<0.001) (**f**). Scale bars, (**a**) 200 *μ*m; (**c**)20 *μ*m; (**d**, **e**) and inset in (**a**) 10 *μ*m

To validate these findings in neurons *in vivo*, in the intact mouse nervous system, we used a transgenic mouse model of tauopathy, THY-Tau22 (hereafter Tau22) mice overexpressing mutant tau protein in the brain.^30^ In 5- to 8-month-old Tau22 animals, phosphorylated tau could be readily detected with AT8 antibody both in the cortex and hippocampus but its levels were moderate in the majority of neurons and only a minor fraction of neurons presented with dense accumulations of phosphorylated tau protein (Figure 4a). Despite that, p-eIF2*α* appeared uniformly upregulated in cells of these brain regions of transgenic animals compared with non-transgenic littermates (Figure 4a,Supplementary Figure 4, ‘no stress’ panels), indicating that even moderate levels of phospho-tau are sufficient to trigger some aspects of mild stress response. Upregulation of p-eIF2*α* as well as GADD34 and CReP proteins in hippocampus was also confirmed by immunoblotting (Figure 4b).

We next induced stress in these mice by applying hyperthermia (heat stress, HS) *in vivo* using a recently developed protocol^31, 32^ (also see Materials and Methods for details). As expected, levels of p-eIF2*α*, GADD34 and CReP increased after HS in the cortex and hippocampus of both WT and Tau22 animals (Figure 4a, Supplementary Figure 4, ‘HS’ panels, Figure 4b). We did not detect significant differences in p-eIF2*α* levels between HS WT and transgenic mice by immunoblotting (Figure 4b). However, a complementary approach, measurement of p-eIF2*α* staining intensity in the cytoplasm, showed its decreased cytoplasmic levels in stressed Tau22 animals as compared with stressed WT mice (Figure 4c).

Hyperthermia in live animals led to SG assembly in neurons as evidenced by the appearance of multiple TIAR-positive and polyadenylated mRNA-positive foci in the cytoplasm of these cells (Figures 4d and e). As predicted from decreased cytoplasmic p-eIF2*α* levels (Figure 4c), Tau22 mice demonstrated significantly reduced number of SG-containing cells in response to HS both in the cortex and hippocampus (Figure 4f).

## Discussion

**Figure 5:**
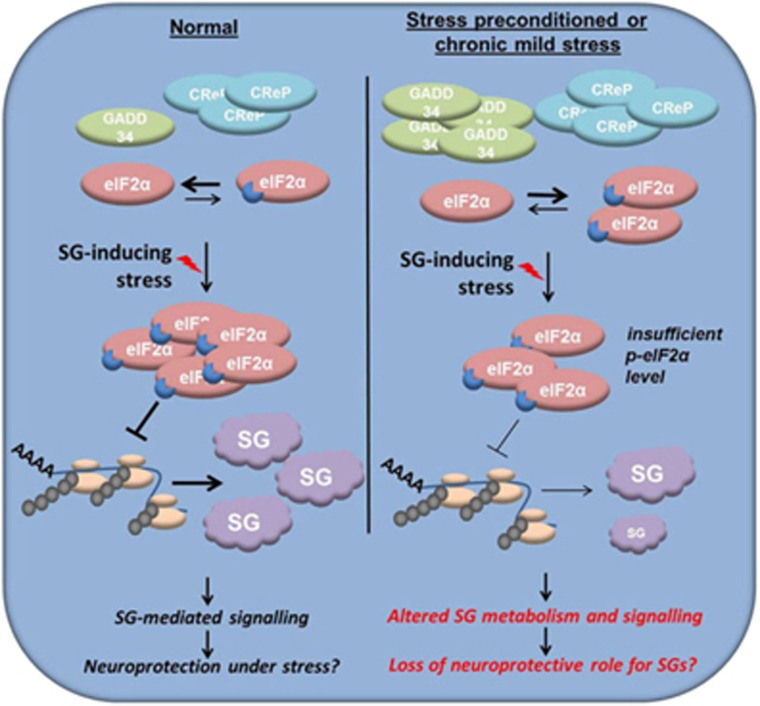
Impaired SG assembly in chronically stressed or stress-preconditioned neurons

Our observations in cultured cells, including neurons, as well as *in vivo*, in a mouse model of tauopathy, strongly suggest that elevated pre-stress p-eIF2*α* levels can be associated with impaired assembly and integrity of SGs. Both mild short-term and chronic stress characterized by p-eIF2*α* upregulation will lead to enhanced activity of its phosphatase complex and hence accelerated p-eIF2*α* dephosphorylation during subsequent strong, SG-inducing stress. As SG formation requires translational shutdown and retaining p-eIF2*α* levels above a certain threshold, early decline eIF2*α* phosphorylation in such preconditioned cells will disable physiologically relevant SG assembly/maintenance during stress and recovery (Figure 5).

Premature recovery from stress and untimely disassembly of SG would have deleterious consequences for any type of cells because of translational maladaptation, altered SG-associated signaling, RNA damage and protein misfolding but could be particularly harmful for neuronal well being. We were able to show that survival of cultured human neurons is negatively affected by stress preconditioning. Tau22 mouse model used in our study is also characterized by increased number of TUNEL-positive neurons in the brain after HS,^32^ and impaired SG assembly may also be contributory in this case. Targeting p-eIF2*α* was shown to be beneficial in several models of neurodegeneration.^18, 33, 34, 35^ The above studies relied on pharmacological inhibition of GADD34 and/or CReP. As, as we showed, elevated levels of both regulatory subunits correlate with disrupted SG assembly, lowering their activity could contribute to neuroprotection in these models by restoration of SG function.

On the other hand, chronic upregulation of p-eIF2*α*, if persists, may also become deleterious. First, prolonged translational repression, which may be associated with increased p-eIF2*α* levels is dangerous for metabolically active and long-living cells such as neurons.^36^ At least one example of a direct link between enhanced phosphorylation of eIF2*α* and development of neurodegenerative changes is known – enhanced translation of BACE1 in the presence of high p-eIF2*α* levels leading to A*β* production and Alzheimer’s disease pathology.^16^ Our study identifies second possible mechanism of toxicity caused by persistently elevated p-eIF2*α* – premature recovery and compromised SG assembly during acute stress. We found that this mechanism is triggered *in vivo*, in a mouse model of Tau pathology, further linking alteration of p-eIF2*α* levels to Alzheimer’s disease pathology. Consistent with this, preventing p-eIF2*α* build-up ameliorated neurodegeneration in a number of mouse models.^15, 17, 19, 37, 38^

It should be noted, however, that while only the consequences of dysregulated p-eIF2*α* phosphatase complex activity for SG assembly/maintenance have been addressed in this study, there are multiple other factors, which would influence this process, the major ones being the activity of eIF2*α* kinases, availability of chaperones, levels of core SG proteins and cytoskeleton integrity.^39, 40, 41, 42^ Therefore, the combination of these factors will eventually define the rate of SG assembly and disassembly in each case.

In our study, we were able to induce SG assembly in neurons of live mice. This *in vivo* model of hyperthermia^31, 32^ is a unique tool to address different aspects of SG formation in the mammalian nervous system and particularly in the nervous system affected by early stages of neurodegeneration.

Altered SG metabolism is of particular importance for ALS and related diseases, and it is still not clear whether their pathogenesis is characterized by enhanced or, on the contrary, attenuated, SGs assembly. However, multiple lines of evidence point to disrupted SG function in these conditions: loss and/or gain of function by several ALS-related proteins, including FUS, TDP-43, angiogenin, C9ORF72-derived dipeptides and hGLE1, negatively affect SG integrity.^43, 44, 45, 46, 47, 48^ The fact that core SG proteins, such as PABP1 and eIF4G, are found accumulated in pathological inclusions in postmortem tissue^49^ also implies a decline of their functional pools and associated deficits in SGs formation. As dysregulation of the above proteins has also been shown to increase cellular p-eIF2*α* levels,^19, 43, 47, 50^ it may result in altered SG assembly via the mechanism described in this study.

In conclusion, our data support the loss of SG function hypothesis in molecular pathogenesis of different neurodegenerative diseases.

## Materials and methods

### Stable cell lines and treatments

CReP KO mouse embryonic fibroblasts (MEFs)^29^ and isogenic WT control MEFs were a kind gift of David Ron (University of Cambridge, Cambridge, UK). SH-SY5Y human neuroblastoma cells and MEFs were maintained in Dulbecco’s modified Eagle’s medium (Invitrogen, Waltham, MA, USA), supplemented with 10% fetal bovine serum, 100x penicillin/streptomycin and 200 mM glutamine. The following compounds and concentrations were used: SA (Sigma, Gillingham, UK): 0.5 mM; MG132 (Calbiochem, Merck, Darmstadt, German): 1 *μ*M and 200 nM; guanabenz (Sigma): 5 and 50 *μ*M; pifithrin *μ* (HSP70 inhibitor): 5 *μ*M (Enzo Life Sciences, Exeter, UK). CReP MISSION esiRNA was purchased from Sigma (EMU020261) and transfected using Lipofectamine2000 (Invitrogen) according to the manufacturer’s instructions.

### Primary mouse hippocampal cultures

Primary cultures of mouse hippocampal neurons were prepared from newborn CD1 mice as described.^8^ Briefly, hippocampi were dissected, digested for 40 min in 0.1% trypsin in HBSS supplemented with 10 mM Hepes and 1 mM pyruvate. After mechanical dissociation in Neurobasal A medium supplemented with 50 U/ml penicillin/streptomycin, 0.2% *β*-mercaptoethanol, 500 *μ*M l-glutamine and 10% horse serum, hippocampi were centrifuged for 5 min at 1500  r.p.m. Pellets were resuspended in fresh medium and plated on poly-l-lysine-coated coverslips. One day after plating, the medium was changed to serum-free medium containing B27 and then changed every second day. All reagents were from Life Technologies.

### Differentiation of human ES cells into motor neuron enriched cultures

The hES H9 cell line was maintained in mTESR2 media (Stemcell Technologies, Cambridge, UK) on Matrigel (Corning, New York City, NY, USA)-coated dishes. Cells were differentiated into motor neurons using published protocols^51, 52^ with modifications. Briefly, confluent hES H9 cell cultures were switched to differentiation medium of the following composition: advanced DMEM/F12 (ADF) supplemented with GlutaMAX, penicillin-streptomycin (all Gibco, Grand Island, NY, USA) and SB431542 (10 *μ*M, Abcam, Bristol, UK). On day 4, purmorphamine (1 *μ*M, Cayman Chemicals, Ann Arbor, MI, USA) and retinoic acid (0.1 *μ*M, Sigma) were added to the above media. On day 8, cells were split in 1 : 2 ratio. On day 16, neural progenitors were enzymatically dissociated using Accutase (Gibco) and plated onto poly-l-lysine Matrigel-coated dishes and cultured in ADF supplemented with GlutaMAX, penicillin–streptomycin, B27 (12587-010) and N2 supplements (Gibco) and BDNF (Miltenyi, Bergisch Gladbach, Germany, 10 ng/ml). On day 23, neurons were re-plated using Accutase onto poly-l-lysine/laminin (Sigma) dishes and coverslips at desired density and cultured in the same media as above, except 50 : 50 mixture of ADF/Neurobasal A (Gibco) was used, until day 40. To assess the number of apoptotic cells, total number of caspase 3-positive cells per was quantified per × 20 magnification field using ‘Analyze particles’ tool of ImageJ software (https://imagej.nih.gov/ij/).

### Immunofluorescence on coverslips and SG analysis

Cells were prepared for fluorescent microscopy as described previously.^53^ Briefly, cells were fixed with 4% paraformaldehyde on ice for 15 min, followed by washes with PBS and 5-min permeabilization in cold methanol. After blocking in 5% goat serum/PBS/0.1% Triton X-100 for 1 h at room temperature, coverslips were incubated with primary antibodies diluted in blocking solution for 1 h at room temperature or at 4 °C overnight. Secondary fluorochrome-conjugated antibody was added for 1 h at room temperature in dark place and nuclei were stained with DAPI. Coverslips were mounted on glass slides, on drop of Immumount mounting media (ThermoScientific, Cramlington, UK). Fluorescent and phase contrast images were taken using BX61 microscope, F-View II camera and Cell F software (all Olympus, Tokyo, Japan). Images were prepared using Adobe Photoshop CS3 (San Jose, CA, USA) or Microsoft PowerPoint 2003 (Reading, UK) software. Cells possessing two or more large (mature) SGs (visualized by anti-TIAR or anti-G3BP1 staining) and total number of cells per a view field (× 100 magnification) were counted in 20 or more randomly chosen fields (total ~200–300 cells per coverslip for SH-SY5Y cells and MEFs and ~400 cells for neurons) and mean ratio value was used for statistics.

### RNA expression analysis

RNA extraction and quantitative real-time PCR were performed as described previously.^8^ Briefly, total RNA from cells was extracted using RNAEasy mini-kit (Qiagen, Manchester, UK) or TRI-reagent (Sigma). First-strand cDNA was synthesized using random primers (Promega, Southampton, UK), and SuperScriptIII or SuperScriptII reverse transcriptase (Invitrogen). Quantitative real-time PCR was run in triplicate on an ABI StepOneTM real-time PCR instrument and data were analyzed using StepOneTM Software v2.0 (Applied Biosystems, Foster City, CA, USA) and the 2-ΔΔCT method with DyNAmo HS SYBR Green supermix and ROX (Invitrogen) as a passive reference dye. cDNA amount for each gene was normalized to that of GAPDH. Primer sequences used were as follows: GAPDH: 5′-TCGCCAGCCGAGCCA-3′ and 5′-GAGTTAAAAGCAGCCCTGGTG-3′ CHOP: 5′-TTAAAGATGAGCGGGTGGC-3′ and 5′-GCTTTCAGGTGTGGTGATGTA-3′ GADD34: 5′-GTAGCCTGATGGGGTGCTT-3′ and 5′-TGAGGCAGCCGGAGATAC-3′ CReP: 5′-TCGGTACAGCGTGACGTTC-3′ and 5′-TGGTCCTTTGCGATCCTCAT-3′ GADD34 (mouse): 5′-GGGTGGTCCAGCTGAGAATG-3′ and 5′-CAGGGGTGCTGGGTTTGTAT-3′.

### Puromycin labeling of newly synthesized proteins

Puromycin at a final concentration of 10 *μ*g/ml was added directly to the media 30 min before lysis. In negative control samples, cycloheximide was added together with puromycin at a final concentration of 10 *μ*g/ml. After several washes in 1xPBS, cells were lysed directly in SDS-PAGE loading buffer. Puromycilated proteins were detected by western blotting using a monoclonal anti-puromycin antibody.

### Analysis of proteins by western blotting

Total cell lysates were prepared by lysing cells on dishes in a loading buffer followed by denaturation at 100 °C for 5 min. SDS-PAGE and detection of proteins were carried out as described earlier.^43^ Quantification of band intensities was performed using ImageJ software and mean intensity for a control sample was taken as equal 1. P-eIF2*α* levels were normalized to those of total eIF2*α*.

### Primary antibodies

Commercially available primary antibodies against the following antigens were used: eIF2*α* phosphorylated at Ser51: rabbit monoclonal, ab32157 (Abcam); total eIF2*α*: rabbit monoclonal, D7D3 (Cell Signaling, Danvers, MA, USA); CReP: rabbit polyclonal, 14634-1-AP (Proteintech, Manchester, UK); GADD34: rabbit polyclonal, 10449-1-AP (Proteintech); FUS: rabbit polyclonal, 11570-1-AP (Proteintech); GFP (Living colors, Clontech, Mountain View, CA, USA); G3BP1: mouse monoclonal, 611126 (BD Biosciences, Oxford, UK); TIAR: mouse monoclonal, 610352 (BD Biosciences); puromycin: mouse monoclonal, clone 12D10 (Merck Millipore, Darmstadt, Germany); cleaved caspase 3: rabbit polyclonal, 9661S (Cell Signaling); AT8, Phospho-Tau(Ser202-Thr205): mouse monoclonal (Merck Millipore); *β*III-tubulin: rabbit polyclonal (Sigma); neurofilament 160/200: mouse monoclonal (Sigma); synaptophysin: mouse monoclonal, 611880 (BD Biosciences); ChAT: rabbit polyclonal (Merck Millipore); beta-actin: mouse monoclonal, A5441 (Sigma). Antibodies were used at 1 : 1000 dilution for all applications.

### Induction of hyperthermia in mice

All of the animal experiments were performed in compliance with and following the approval of, the local Animal Resources Committee (CEEA 342012 on 12 December 2012), standards for the care and use of laboratory animals, and the French and European Community rules. Age-matched 5- to 8-month-old mice were subjected to transient hyperthermic stress as previously described.^31^ Briefly, the mice were anesthetized using xylazine (20 mg/kg) and ketamine (100 mg/kg) and maintained in a 37 °C environment for 30 min to avoid anesthesia related hypothermia. The mice were then maintained at 37 °C (control) or HS by being placed in an incubator containing ambient air heated to 44 °C for 20 min. The rectal temperature of the mice was monitored every 10 min and did not exceed 41 °C.

### Preparation of hippocampal cytosolic fractions

Mouse tissues were harvested in ice-cold buffer A (10 mM HEPES, pH 7.9, 1.5 mM MgCl, 10 mM KCl, 0.15% NP-40) supplemented with protease inhibitors (Complete Mini, Roche, Burgess Hill, UK) and phosphatase inhibitors (125 nM okadaic acid and 1 mM orthovanadate). The tissues were mechanically homogenized using a 50-ml all-glass homogenizer on ice and centrifuged at 100 g for 1 min. The supernatant was collected, and a second homogenization was conducted. The supernatant was collected as the cytoplasmic fraction after centrifugation at 1000 *g* for 10 min. The protein concentration was determined using a BCA kit.

### Immunohistochemistry, RNA-FISH and quantitative analyses in mouse brain

Mouse brains were fixed, embedded in paraffin wax, cut 8 *μ*m thick sagittal sections and mounted on poly-l-lysine-coated slides. For detection of polyadenylated RNA by RNA-FISH, deparaffinized and rehydrated mouse brain samples were incubated at 37 °C overnight with 10 *μ*M Cy5-labeled oligo(dT)30 probe diluted in hybridization buffer (2x SSC, 25 % formamide, 10 % dextran sulfate, 0.005 % BSA, 1 mg/ml yeast tRNA). After washing in 2x SSC and 1x PBS, nuclei were counterstained with DAPI. Immunostaining was performed as described previously.^54^ For detection of p-eIF2*α*, Abcam E90 antibody was used. The same microscope, camera and software as for analysis of cultured cells were used. Neurons containing dense TIAR-positive granules were counted in seven non-overlapping areas in the cortex and hippocampus of 5- to 8-month-old animals subjected to hyperthermia (three WT and three Tau22 mice), with >100 neurons counted per animal. To estimate p-eIF2*α* levels in the cytoplasm of hippocampal neurons, fluorescence intensity was measured in a 10 × 10-pixel square in neuronal cytoplasm and mean value for WT mice was taken as equal 1.

### Statistics

Non-parametric Kruskal–Wallis ANOVA and Mann–Whitney *U*-test were used to assess significance of the difference between groups. Bar charts represent mean±S.E.M., *n* corresponds to the number of biological replicates.
