# Supplementary material for: Chronically stressed or stress-preconditioned neurons fail to maintain stress granule assembly
Source: Cell Death Dis. 2017 May 11;8(5):e2788–. doi: 10.1038/cddis.2017.199 (PMC5520719; doi:10.1038/cddis.2017.199)
Supplement: Supplementary Figures [file cddis2017199x1.doc]

**Chronically stressed or stress-preconditioned neurons fail to maintain stress granule assembly**

Tatyana A. Shelkovnikova, Pasquale Dimasi, Michail Kukharsky, Haiyan An, Annamaria Quintiero, Claire Schirmer, Luc Buée, Marie-Christine Galas and Vladimir L. Buchman

**Supplementary Figures**


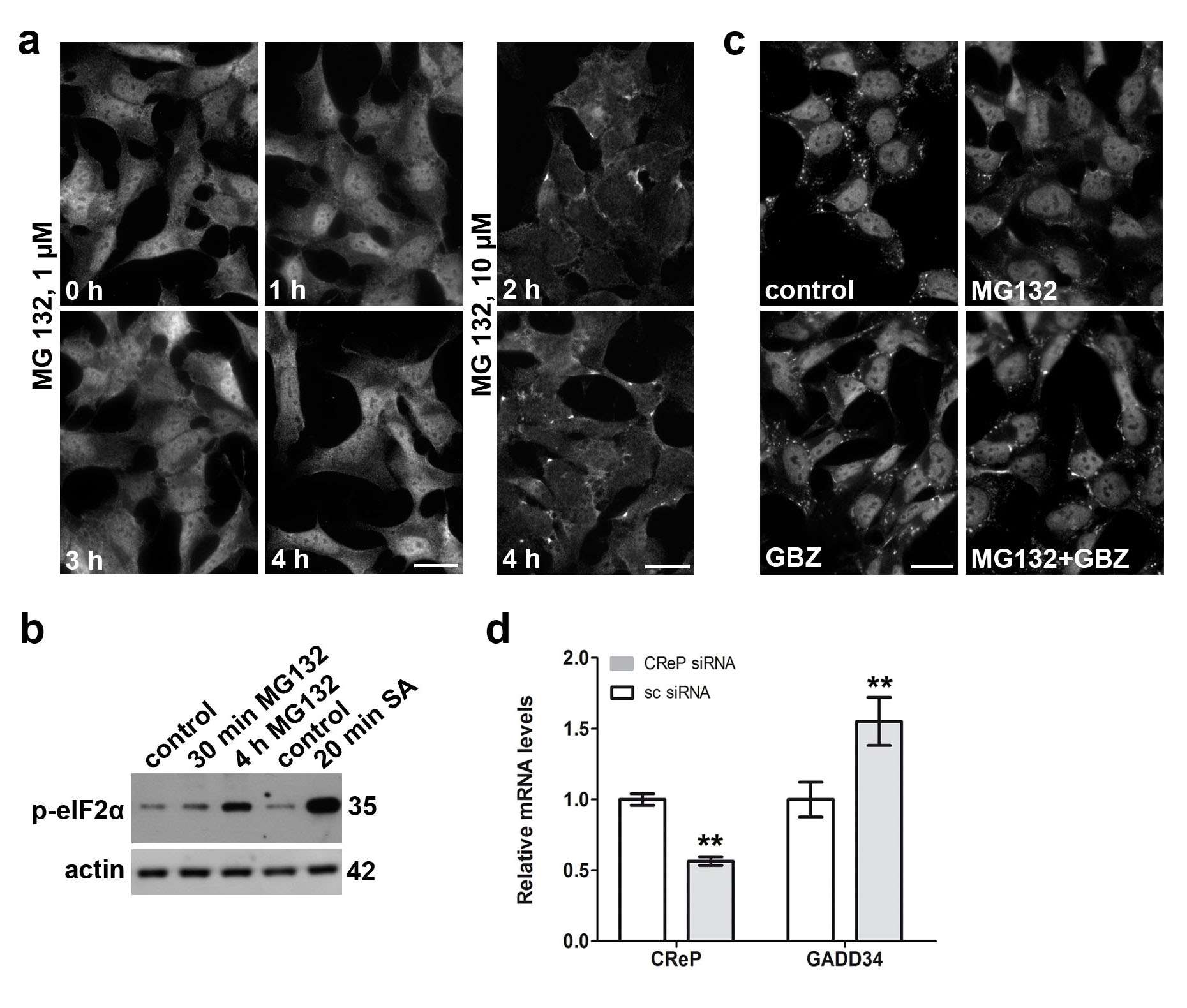


**Figure S1. Characterisation of the effects of MG132 treatment and CReP knockdown on neuroblastoma cells. (a,b)** Treatment with 1 µM MG132 does not induce SG formation and moderately increases p-eIF2α levels in neuroblastoma cells. Cells were treated with MG132 for the indicated times and stained for a SG marker TIAR (a) or analysed by Western blot (b). Representative images and Western blot are shown (a). Note that consistent with previous reports, 10 times higher MG132 concentration induces SG formation in SH-SY5Y cells. **(c)** Guanabenz (GBZ) rescues SG assembly in MG132-pretreated cells. Cells were treated with MG132 and/or GBZ and stressed with SA for 20 min. Representative images are shown. **(d)** Downregulation of CReP by siRNA in neuroblastoma cells leads to compensatory increase of GADD34 mRNA (n=6, ** - p<0.01).

**
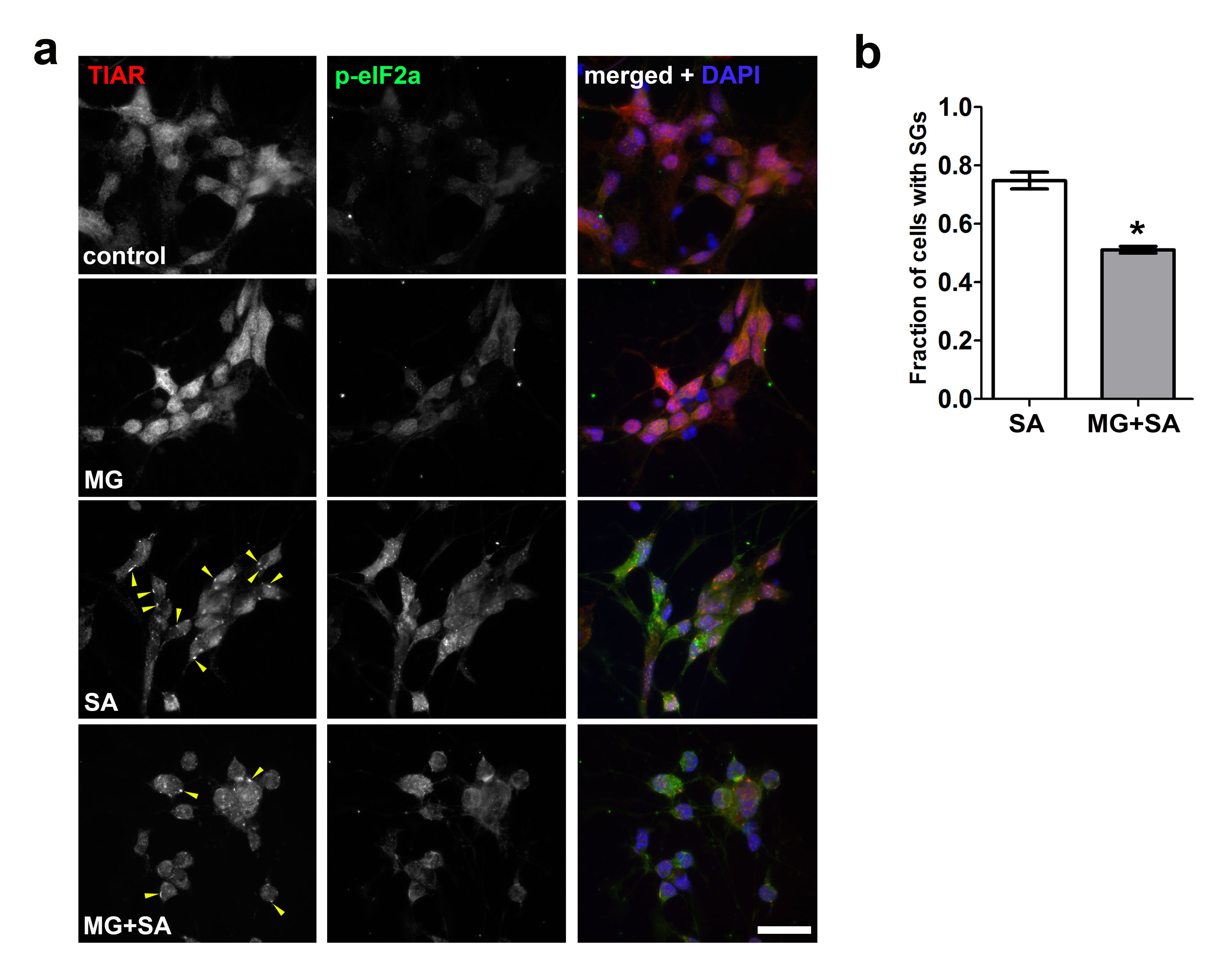
**

**Figure S2. Induction of p-eIF2α and SGs in cultured mouse hippocampal neurons. (a, b)** The level of p-eIF2α is increased in mouse primary hippocampal neurons (DIV7) in response to MG132 and SA (a), and SG assembly is impaired in MG132-treated cells (a,b). Cells were pretreated with MG132 (200 nM for 4 h) or left untreated, and SA was added for 70 min. Arrowheads indicate SGs. Fraction of cells with TIAR-positive SGs was quantified in ~200 cells in each of the four independent experiments (* - p<0.05). Scale bar, 10 µm.


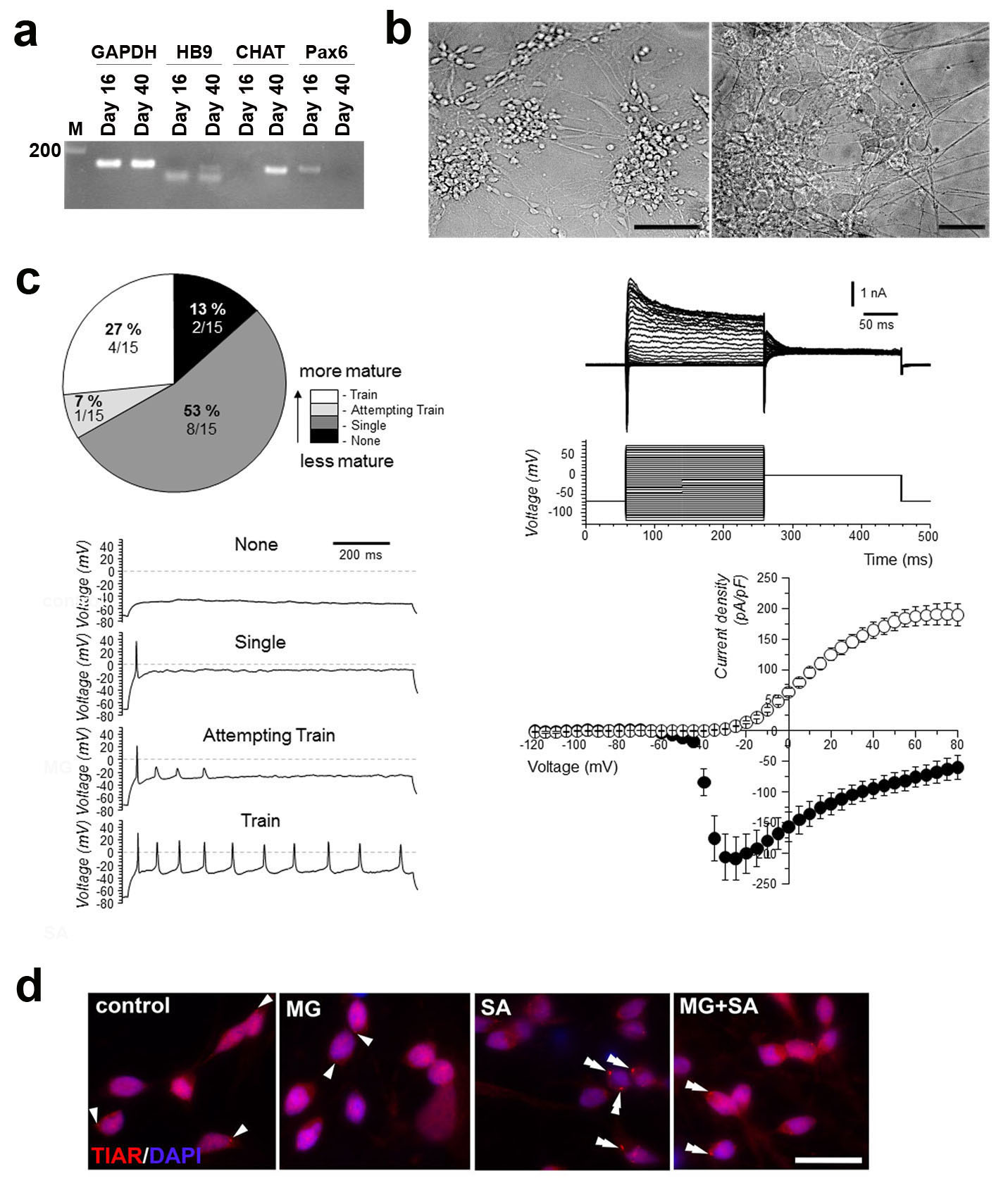


**Figure S3. Characterisation of hESC-derived neurons used in the study. (a)** RT-PCR analysis of expression of markers typical for neural precursors and mature motor neurons on days 16 and 40 of differentiation. **(b)** Phase contrast images of cultures of day 40 neurons. **(c)** Electrophysiological recordings of day 40 neurons. Left: Pie chart showing the proportion of day 40 neurons exhibiting trains of induced action potentials (iAP-train), attempted trains of action potentials (iAP-Attempted Train), single induced action potential (iAP-single), or no induced action potentials (iAP-None). Current-clamp recordings when the neurons were held at around -70 mV and to 1s injected currents of +80 pA exemplifying the model activity; expanded time-base sections of traces are shown below the traces. Right: Upper panel -exemplar families of whole-cell currents evoked by the voltage activation/inactivation protocol. Medium panel represents voltage step protocol. Lower panel - mean current density ± SEM vs. voltage plots derived for maximum voltage-activated Na+ (filled circles) and steady state K+ currents (open circles) derived from traces exemplified in upper panel. **(d)** TIAR-positive dots are present in the soma of human cultured neurons in the absence of stress. Arrowheads point to TIAR-positive dots observed in naïve cells, double arrowheads point to SA-induced SGs. Scale bars, b – left, 200 µm and right, 10 µm; d – 10 µm.

The following techniques were used for electrophysiological studies:

Patch-clamp - voltage and current recordings were made using conventional patch-clamp in the whole-cell configuration [7] employing Axopatch 200B amplifier interfaced to a computer running pClamp 9 using a Digidata 1322A A/D interface (Molecular Devices, Sunnyvale, CA, U.S.A.). All electrophysiological studies were performed at a controlled room temperature of 22 ± 0.5 °C. Recordings were digitized at 10 kHz and low-pass filtered at 2 Hz using an 8-pole Bessel filter. The standard bath solution contained (in mM): 135 NaCl (Fisher Scientific UK Ltd, Loughborough, Leics., UK), 5 KCl (Fisher), 1.2 MgCl_2_ (Sigma-Aldrich), 1.25 CaCl_2_ (Sigma-Aldrich), 10 D-glucose (Fisher), 5 N-2-hydroxyethylpiperazine-N'-2-ethanesulfonic acid (HEPES, VWR International); pH was adjusted to 7.4 using 5 M NaOH. The standard pipette solution contained (in mM): 117 KCl, 10 NaCl, 11 N-2-hydroxyethylpiperazine-N'-2-ethanesulfonic acid (HEPES), 2 Na_2_-ATP (Sigma-Aldrich), 2 Na-GTP (Sigma-Aldrich), 1.2 Na_2_-phosphocreatine (Sigma-Aldrich), 2 MgCl_2_, 1 CaCl_2_ and 11 ethylene-glycol-tetra-acetic acid (EGTA, Fisher); pH was adjusted to 7.2 with KOH.

Mean resting membrane potential (Vm) of the neurons were determined during 120 s gap-free recording periods in current clamp mode (I = 0 pA). Once Vm had been recorded, current was injected to hyperpolarize Vm to ca. -70 mV before 1 s current injection steps were imposed (from -10 pA to +180 pA with increment of 10 pA) in order to record induced action potential (iAP) activity, which was coded as: iAP-none (no significant excursions from baseline during injection); iAP-single (a single excursion which overshoots 0 mV), iAP-attempted train (several excursions but only 1 overshoots 0 mV), and; iAP-train (several excursions, at least 2 of which overshoot 0 mV). Where iAP trains were recorded, a spike frequency analysis was performed. Input resistance was measured from the voltage difference induced by the -10 pA current step. Spike analysis was performed on the first iAP using Clampfit 9; threshold was determined as the peak of the 3^rd^ differential of voltage with respect to time during the upstroke of the action potential and all other parameters are as defined extensively elsewhere.

Na^+^ currents were recorded using a standard voltage-step protocol (holding potential of -70 mV followed by 80 ms steps from -120 to +80 mV in increments of 10 mV). For Na^+^ current inactivation curves, cells were stepped for 200 ms to pre-pulse voltages of between -120 and +80 mV in 5 mV increments before being stepped for 200 ms to the test potential of 0 mV. Cell capacitance and series resistance were measured and compensated; series resistance was compensated 60-90%. Pipette resistances were 8-10 MΩ when filled with the pipette solutions.

For activation and inactivation curves, conductance (G) was calculated by dividing current by the appropriate driving force, (V_c_ – E_Na_), where V_c_ = command potential, and E_Na_ = +66.7 mV. G/G_max_ was plotted against voltage and fitted with a Boltzmann equation using an iterative fitting routine in Microcal Origin:

G/G_max_ = 1/[1 + exp (V_0.5_ – V_c_)/k]

where G_max_ is the extrapolated maximum conductance, V_0.5_ is the voltage corresponding to half the maximum conductance and k is the slope factor.


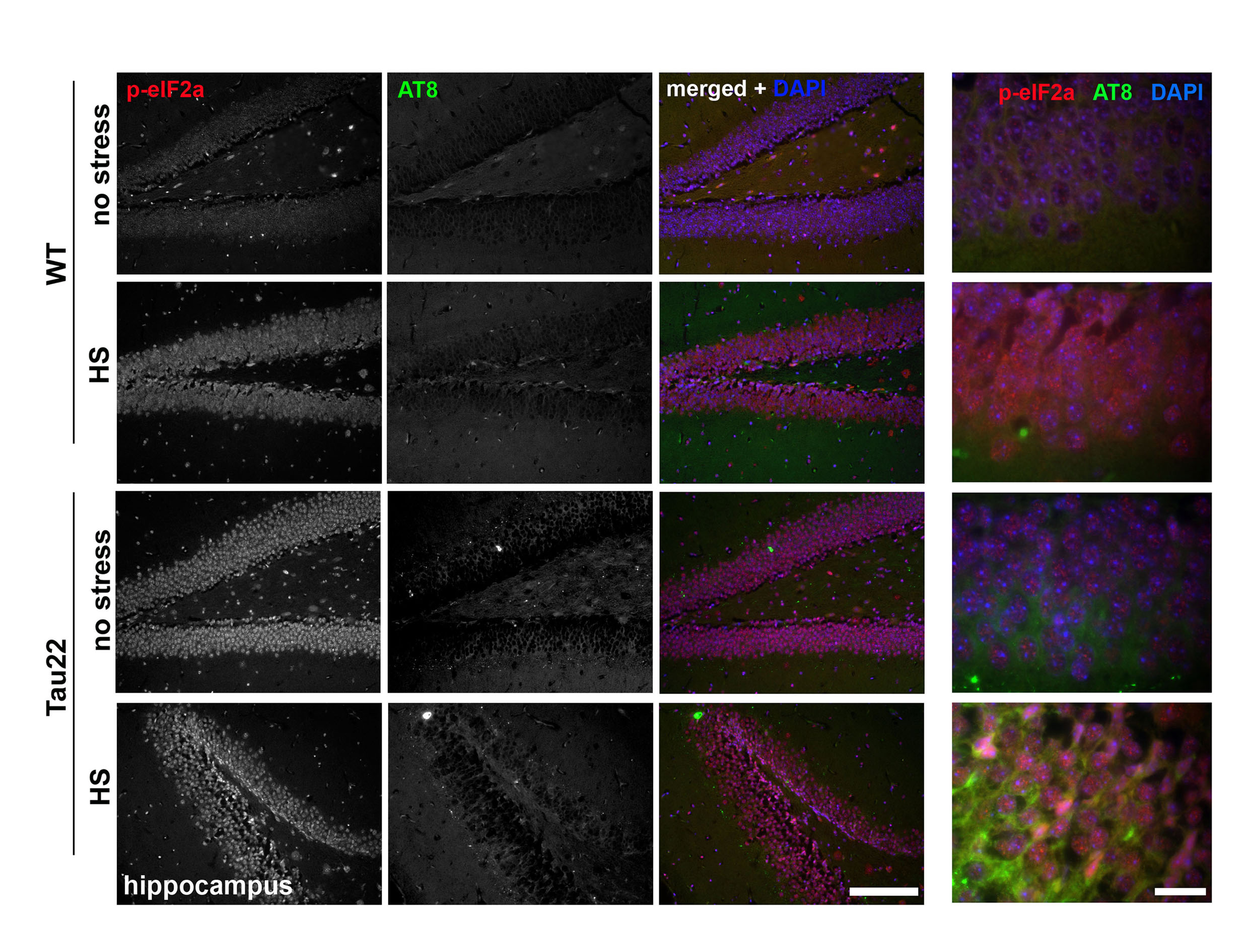


**Figure S4. Elevated p-eIF2α in the hippocampus of Tau22 transgenic mice and before and after stress.** p-eIF2α is upregulated in the hippocampus of 6-month old Tau22 transgenic mice and heat stress (HS) further increases its levels. Tau pathology was visualized using phospho-specific tau (AT8) antibody. Scale bars, 200 µm for left (low magnification) panels and 10 µm for the right column (high magnification).
